# mHealth to Improve Experience, Adherence to Pharmacological Treatment, and Positive Mental Health in Patients Diagnosed With Femur Fractures: Protocol for a Quasi-Experimental Study

**DOI:** 10.2196/45856

**Published:** 2023-04-28

**Authors:** Gemma Marcos Anton, Montserrat Puig Llobet, Teresa Lluch Canut, Maria Aurelia Sanchez Ortega, Mercè Piazuelo Pont, MªCarmen Moreno-Arroyo

**Affiliations:** 1 Department of Traumatology Hospital Clínic de Barcelona Barcelona Spain; 2 School of Nursing Faculty of Medicine and Health Sciences University of Barcelona L'Hospitalet de Llobregat (Barcelona) Spain; 3 Department of Public Health Nursing University of Barcelona L'Hospitalet de Llobregat (Barcelona) Spain; 4 Department of Mental Health Nursing University of Barcelona L'Hospitalet de Llobregat (Barcelona) Spain; 5 Department of Maternal and Child Health Nursing University of Barcelona L'Hospitalet de Llobregat (Barcelona) Spain; 6 Nursing and Occupational Therapy School Universitat Autònoma de Barcelona Terrassa Spain; 7 Department of Fundamental Care and Medical-Surgical Nursing Faculty of Medicine and Health Sciences University of Barcelona L'Hospitalet de Llobregat (Barcelona) Spain; 8 Department of Nursing Research Group (GRIN) Bellvitge Biomedical Research Institute (IDIBELL) L'Hospitalet de Llobregat (Barcelona) Spain

**Keywords:** education, patient experience, medication adherence, mental health, nurse, mHealth, mobile health, protocol, prognosis, pharmacological treatment, femur fracture, femur, diagnosis, quality of care, care, psychological, efficacy, training, pathology

## Abstract

**Background:**

Considering the prognosis of femur fractures worldwide, the ageing of our society, and the problems in adherence to treatment found in these patients, it is believed that mobile health can have a positive impact on the process and quality of care.

**Objective:**

We aim to evaluate the effectiveness of a pharmacological educational nurse intervention with Myplan app with regard to knowledge, adherence to pharmacological treatment, and positive mental health of patients with femur fractures.

**Methods:**

A nonrandomized, quasi-experimental study will be carried out with a pretest-posttest control group. It will include 278 older patients diagnosed with femur fractures, with a Glasgow Coma Scale of 15 and access to mobile devices. Patients with psychological pathologies and cognitive impairment or patients treated in isolation will be excluded. Study variables are as follows: sociodemographic variables (AdHoc Form), patient experience (Patient Experience Questionnaire-15), adherence to pharmacological treatment (Morisky-Green questionnaire), and positive mental health (Positive Mental Health questionnaire). The measurements will be taken 24 hours after admission, upon discharge, and 25 days after discharge.

**Results:**

Enrollment commenced in October 2022. Data collection will be completed in April 2023.

**Conclusions:**

The results of this study will offer evidence of the effectiveness of a pharmacological educational nurse intervention by means of a free smartphone app. If its efficacy is demonstrated and the results are acceptable, it could mean an improvement in the care of patients with femur fractures, and this technology could be used to guide other training interventions in patients with other pathologies.

**Trial Registration:**

ClinicalTrials.gov NCTT05669040; https://clinicaltrials.gov/ct2/show/NCTT05669040

**International Registered Report Identifier (IRRID):**

DERR1-10.2196/45856

## Introduction

### Overview

The estimated amount of femur fractures by 2050 is 6 million worldwide. Due to the increase in life expectancy, the incidence of femur fractures in people older than 65 years is close to 4-6 cases per 1000 inhabitants per year in Spain, Catalonia, being the region with the highest incidence of femur fractures [1]. It has been shown that the population experiencing this type of fracture exhibits a high degree of complexity, causing an increase in morbidity, mortality, and subsequently, health care expenditure [2]. It is, therefore, necessary to establish an adequate health care quality, where the affectivity of the treatment and the safety of the patient are guaranteed [3].

There is evidence of the relationship between a good patient experience and both direct and indirect clinical results, which leads to better prognosis, faster discharges, and lesser use of medication [4,5]. Another important factor is the relationship between positive mental health and capacity for self-care. The more positive patients’ mental health is, the better their ability for self-care will be, and vice versa [5-7]. Another key factor, as the literature indicates, is health education for patients regarding their illness and its treatment. The more knowledge they have, the better their health and ability for self-care will be [8,9]. We usually find education studies focused on patients diagnosed with chronic diseases [10], but we rarely find studies that evaluate the effectiveness of education and information in relation to medication adherence.

When informing patients about medication, the term “10 rights” [11] regarding the safe administration of medication should also be mentioned—inform the patient about the medication that is being used, how it must be taken, what it is being used for, and what secondary effects or adverse reactions may derive from its use. The importance of this point stands out in two aspects: (1) the patient’s health education regarding all the medications they take, so they understand everything related to the care of their illness; and (2) the potential detection of errors on the patient’s part, acting as the last filter just before the administration of the drug by the nurse. This point is so important that not informing patients is considered a medication error in some studies [12].

Prescription of medication is one of the most common interventions in clinical practice [13], and it has a significant repercussion in the time used by nurses in administrating medicines, which takes up a substantial part of their workday. Cleveland Hospital applies one of the innovative strategies in administration of medicines, named the Ask3/Teach3 initiative [14]. In this strategy, the patient is informed about the medicine, but its secondary effects are also inserted into that same conversation with the patient.

Studies in medical literature describe that the introduction of educational tools related to medication in health care organizations is linked to a decrease in hospitalization, admissions into emergency rooms, and readmissions [15]. Patients nowadays have a very close relationship with technology, as has been shown in various published studies that prove the use of apps improves health management [16,17]. Mobile health (mHealth) has opened the doors to new forms of relationships between nurses and patients, providing numerous benefits for both parties with regard to accessibility and optimization of time in office and improvement of therapeutic adherence. The mass use of web-based devices by patients older than 65 years (ie, the population sector with the highest probability of requiring medical care) has had a very positive influence in this regard [18].

It is described that there is a lack of adherence due to the complexity of the treatment, its secondary effects, lack of trust in the treatment, and the relationship between health care professionals and patients [19]. The lack of adherence can lead, firstly, to a lack of treatment effectiveness, and secondly, to a potential increase in the secondary effects of the treatment, a problem that affects 50%-60% of patients with chronic diseases. It is estimated that 20%-50% of patients do not take their medication as prescribed [20]. The index of medication adherence in these patients is only 50% [21]; therefore, improving medication adherence would result in a decrease in morbidity, mortality, and expenditure in medical care [22]. Treatment adherence is also linked to patients’ emotional well-being, the mental strength with which they face the situation, and ultimately, their level of positive mental health [23,24]. It has been observed that interventions are needed to improve lack of treatment adherence [25]. No studies have been found in medical literature on the subject of pharmacological adherence and patients with femur fractures.

One of the tools developed to complement patient education is mHealth, described by the World Health Organization (WHO) in its 2015 Global Observatory for Health report (reviewed in 2016) [26] as the use of mobile devices, such as smartphones, personal digital assistants, and patient monitoring devices, in medical practice and public health.

The WHO believes that mHealth can contribute to achieving coverage of health care at a global level, making it accessible to remote populations and communities with health service deficits. This is one of the strategic lines of the government of Catalunya. At the same time, the WHO recognizes that it is not very costly to supply these areas with mobile technology infrastructures [27].

Based on this evidence, this study proposes to respond to the needs raised so far, with the development and evaluation of a pharmacological educational nurse intervention based on a smartphone app (MyPlan app) and with regard to knowledge, pharmacological treatment adherence, and positive mental health of postoperative patients with femur fractures.

### Objectives

The primary objective of this study is to evaluate the effectiveness of a pharmacological educational nurse intervention with MyPlan app in the experience of medical assistance, pharmacological treatment adherence, and positive mental health of patients with femur fractures at the Hospital Clínic de Barcelona. We also intend to compare the pre-post intervention effectiveness in the study variables 24 hours after admission, upon discharge, and 25 days after hospital discharge.

## Methods

### Study Context

This study aims to test the nurse intervention with the added support of mHealth by creating personalized medical care and a 3-stage follow-up plan to improve the patient's experience, adherence, and positive mental health.

### Scope of Study

This project estimates the application of this approach in the Institute of Traumatology and Orthopedics of the Hospital Clínic de Barcelona between October 2022 and April 2023.

### Design

This protocol describes a nonrandomized, quasi-experimental study with a pretest-posttest control group.

### Participant Selection and Recruitment

Eligible participants will be identified in the hospitalization unit through the observation of a census by the principal investigator (PI) with a nonprobabilistic sample (according to the patients’ willingness to participate).

The sample calculation will be made by taking into account the total number of patients treated in the traumatology service for a femur fracture diagnosis during 2020. Approximately 494 patients are admitted annually with a femur fracture diagnosis. GRANMO software (version 7.12; IMIM Medical Informatics Unit) will be used with a 95% CI and a maximum indeterminacy of *P*=.5, accounting for a 15% loss, for a final recommended sample of 278 subjects to ensure a sufficient sample size for the analysis of the different variables.

### Inclusion and Exclusion Criteria

The criteria for inclusion are as follows: (1) femur fracture diagnosis, (2) 18 years of age or older, (3) knowledge of Spanish language, (4) 24-hour admission into the traumatology unit, (5) awareness level of 15 on a Glasgow scale, (6) access to mobile devices, (7) ability to participate in the pilot study, and (8) ability to provide written informed consent personally or to obtain consent via a legal representative. Criteria for exclusion are as follows: (1) presence of psychological pathologies or cognitive impairment and (2) treatment in isolation during hospitalization.

### Ethics Approval

This study has been approved by the Regional Health Research Ethics Committees of the Hospital Clínic de Barcelona (HCB/021/0341).

All questionnaires are confidential, and all patients must sign the informed consent according to the current Spanish legislation. In compliance with the current law on data protection and guarantee of digital rights, no data will be shared without express authorization, preserving patients’ confidentiality and anonymity of their identity at all times. Their participation will be voluntary, and their anonymity will always be preserved. This will be achieved by anonymizing the names or any personally identifiable information.

### Intervention

**Figure 1:**
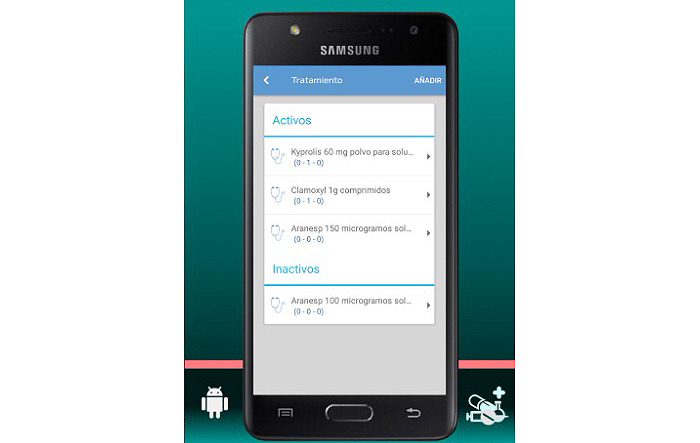
MyPlan app; medication plan.

**Figure 2:**
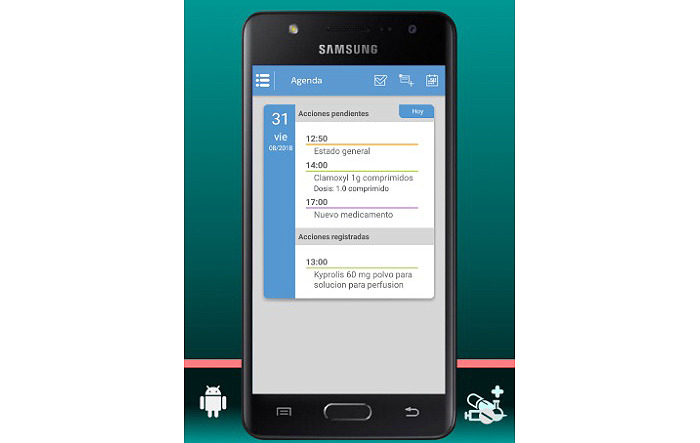
MyPlan app; daily calendar.

**Figure 3:**
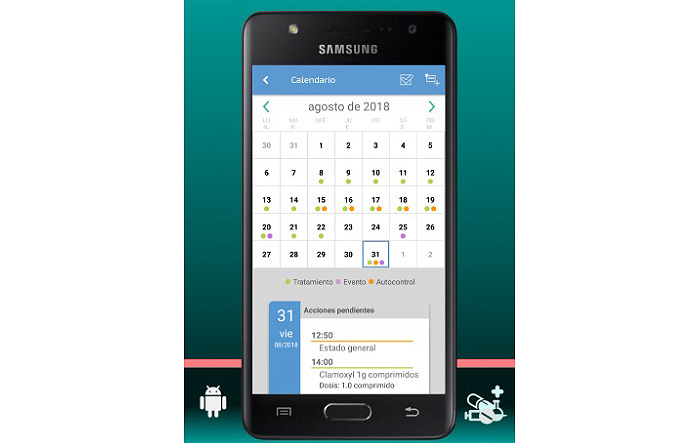
MyPlan app; monthly calendar.

**Figure 4:**
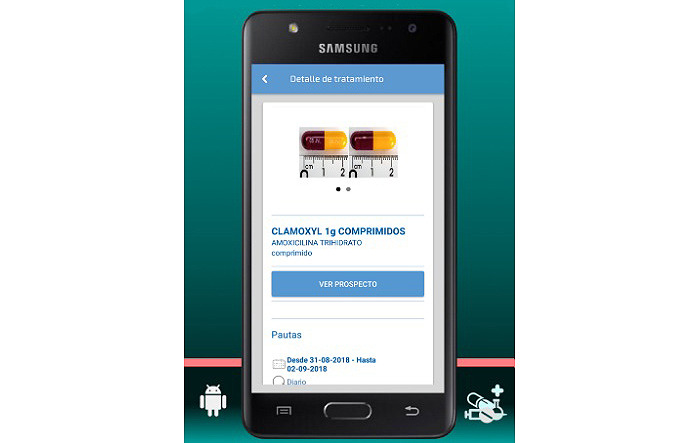
MyPlan app,; individual medication.

The final app is named MyPlan. it is based on a platform developed by the nonprofit organization Trilema, with the support of Amgen laboratory. The app is user-friendly and intuitive, and it allows patients to see their updated medication plan on their mobile phones at all times in a safe and private environment (Figure 1). One of its main functions is the daily (Figure 2) and monthly (Figure 3) calendar, and it also features reminders and allows adding programmed activities (eg, medication times, check-ups, and appointments). MyPlan is also a tool that supports treatment, allowing patients to plan and validate individual medication intake (Figure 4).

**Figure 5:**
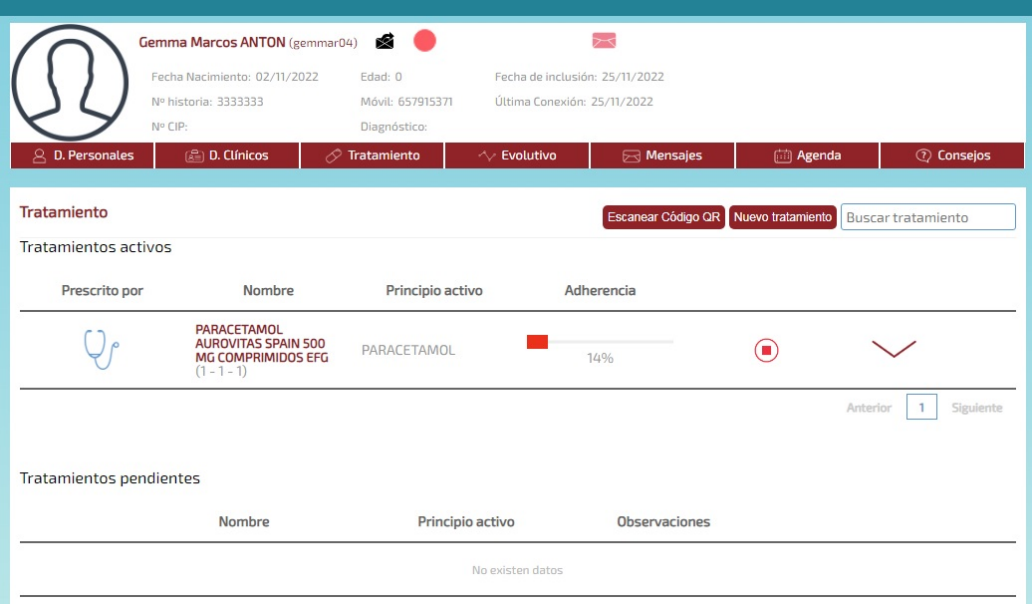
MyPlan app; private area.

To ensure that the intervention works as expected and patients are able to access the app, the PI has a private area in MyPlan (Figure 5), where every individual patient is registered with a personal username and password. The PI will also add their individual medication plans so that patients are able to view them on the app.

**Figure 6:**
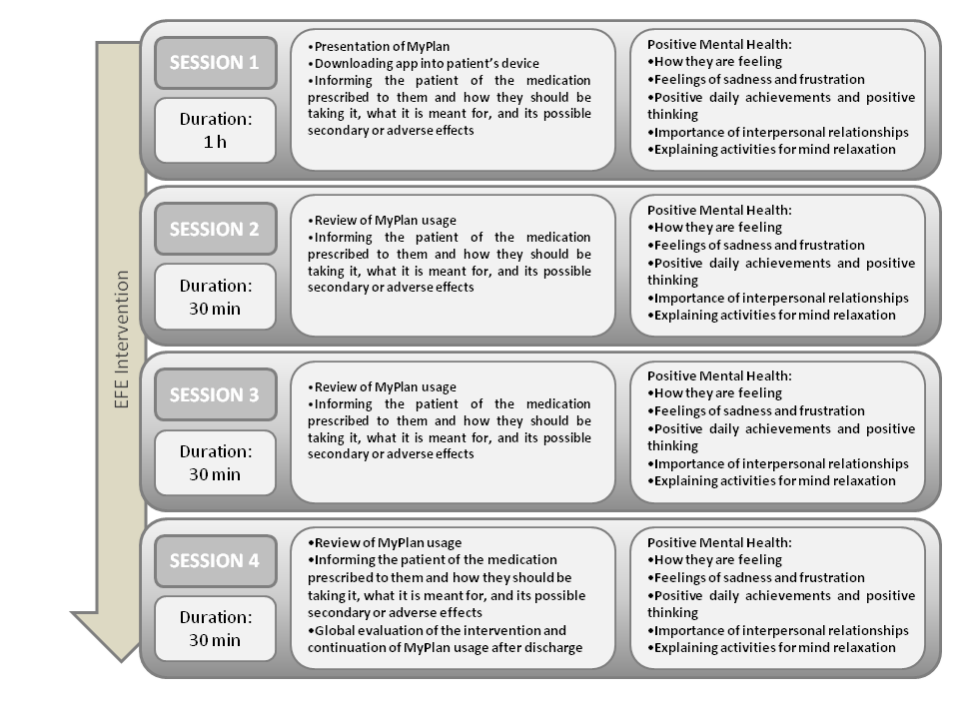
The nursing pharmacological education intervention (EFE).

The nursing pharmacological education intervention will be performed by a single nurse (an employee of Hospital Clínic de Barcelona) and the PI. This intervention consists of 4 sessions (Figure 6). First, the nurse will introduce themselves to the patient as the professional who will carry out the training session. Subsequently, patients will be informed about the intervention and its objectives. They will be introduced to MyPlan app, which will be downloaded on their smartphone, and a username and password will be generated for them (30 minutes). Then, the prescribed treatment and any questions from the patient about it will be discussed and reviewed (20 minutes). Finally, the session itself will be evaluated (10 minutes), using the Morisky-Green scale, as well as the PI’s review of the patient’s use of MyPlan during the admission, making sure they have pressed “OK” at the time they were taking the medication according to the prescription.

Positive mental health will be promoted in every session by giving patients the opportunity to explain how they are feeling, telling them that it is normal to feel sadness and frustration during hospitalization, but also encouraging them to think about the small daily achievements like decrease in pain or improvement in mobility. If the negative feelings persist, the patient will need to ask for help. The nurse will constantly encourage the patient to try to maintain a positive mindset and think that the final outcome will be good. The patient must value the positive things they have in life and cultivate interpersonal relationships. They will receive help in trying to find activities that will help them achieve a state of mental relaxation. This action will be evaluated by using the Positive Mental Health questionnaire.

The patient's experience will be measured with the Patient Experience Questionnaire-15. These tools have been added into MyPlan, making it agile to fill them out and export their data.

### Procedure

Following the inclusion and exclusion criteria defined in the study, the patient will be requested to answer the different questionnaires described in the “measurement tools” section.

The PI will download the MyPlan app for the patient and explain its function to them. The PI will also manually transcribe the treatment that has been prescribed for the patient’s hospitalization time. The medication that must be administered to the patient and the medication schedule will be added into the app. The PI will also carry out a basic training session for each medicine prescribed to the patient, including what the drugs are for and their possible adverse effects.

During hospitalization, the same procedure will be repeated every day from Monday to Friday. A minimum of 4 sessions, as described above, will be held to educate the patient and improve their knowledge in pharmacology and autonomy upon discharge. The PI will maintain an individual registry in a Microsoft Excel spreadsheet that will contain all adverse drug reactions notified in the patient’s clinical history. These data will be extracted from the information in the Systems Applications and Products in Data Processing bed chart (there is a shortcut icon for adverse drug reactions notifications), which is filled in by the medical professionals in charge of any given patient during hospitalization when applicable. As soon as the patient has been deemed fit to be discharged, the treatment prescribed for the time after discharge will be explained to them using the app (we will restrict the explanations to those pertaining to the treatment specified in the discharge report). It will be explained to them that they can use the app during the 30 days following discharge, as long as their primary care doctor does not make modifications to the treatment. For this reason, the patient’s history will be reviewed daily by the PI to make sure there have been no consultations that have resulted in a change in the medication; 25 days after discharge, the PI will contact the patient via the MyPlan app to ask them to fill out the questionnaires and remind them that the study will end 5 days later (ie, 30 days after discharge). A message will be sent to the patient via the app 30 days after discharge to thank them for participating in the study, and their access to the app will be blocked, considering that the intervention has concluded.

### Demographics and Baseline Functioning

Background data about the participants will be collected in the first session of the nursing pharmacological education intervention with the objective of evaluating any significant differences in the results after completion of the test. This background data will include age, gender, nationality, level of education, cohabitation status, caregiver, treatment, time of hospitalization, number of previous hospitalizations, evaluation of own health, and adverse effects.

### Pilot Study

A pilot study will be carried out for the duration of a month with 20 subjects. The objective will be to evaluate the questionnaires’ complementarity, clarity, and understandability. The patients selected for the pilot study will meet the criteria for inclusion. Once the pilot has concluded, modifications to the questionnaires will be made if necessary. The possibility to create a leaflet for the handling of the MyPlan app and the time frame for patient assistance via the app will be discussed.

### Measurement Tools

The following measurement tools have been validated:

- Patient Experience Questionnaire-15, adapted and validated for Spanish language [28,29]: this questionnaire will allow us to explore patients’ perception with regard to the educational process and their participation in the decision-making processes during their hospitalization.
- Morisky-Green Questionnaire [30]: this questionnaire will allow us to evaluate patients’ treatment adherence. It is comprised of 4 binary questions that will reflect the degree of compliance in patients as well as information regarding the causes of noncompliance.
- Positive Mental Health Questionnaire [31]: this scale evaluates mental health from a positive viewpoint. It is comprised of 39 items, which are distributed across 6 categories: personal satisfaction, prosocial attitude, self-control, autonomy, problem-solving and self-actualization, as well as interpersonal relationship skills. Their measurement is made via Likert-type answers with the following range: “always or almost always,” “frequently,” “sometimes,” and “never or almost never.” It is validated among the general population in favorable psychometric values, with global reliability of 0.88 and variance of 45%.
- Forms for collection of sociodemographic and clinical variables.

### Data Analyses

The statistical analysis will be made using Stata (version 15; StataCorp). It will consist of obtaining the differences between different data collections (24 hours after admission, upon discharge, and 25 days after discharge) for every variable considered in this project as a performance measurement of the intervention. Efficacy, effectiveness, and efficiency indicators will be based on the study of change from before the intervention to after the intervention (ie, before the intervention; upon discharge; and the last control, 25 days after discharge at home). These will be based on relative indexes (ie, change percentages) and reliable change indexes (ie, Jacobson-Truax coefficient as well as alternative indexes, such as Tsu, Hagerman-Arrindell, and Speer-Greenbaun indexes). The difference in quantitative measurements in different phases will be based on factorial analysis (ANOVA). These will provide the main effects of change for all different phases. They will also provide the reliability intervals of the averages and the differences of proportion as measures to be assessed, as well as that of standardized indexes, such as Cohen *d*.

## Results

Enrollment was initiated in October 2022 and will be completed in April 2023.

The main contribution of this protocol is a detailed description of a nursing intervention with the support of a smartphone app that will generate knowledge about patients’ experiences, their adherence, and their positive mental health. The protocol is registered with ClinicalTrials (NCTT05669040).

## Discussion

### Expected Findings

One of the strong points of this study is the linear evaluation of the intervention (ie, 24 hours after admission, upon discharge, and 25 days after discharge), which can demonstrate the effectiveness of assisting the patient throughout the process. Another important finding may be related to the impact of the intervention on the quality of care because it could contribute to early detection and prevention of medication errors, minimizing both the associated complications that these patients may have and hospital re-admissions. Finally, another potential strength of the study is that the intervention can be extrapolated to different specialties in the hospital, which would contribute to the improvement of care for all patients.

The main result will be to generate a change in the medication administration process, implementing the nursing pharmacological education intervention. The secondary result will be related to the patient's hospital experience, the increase in treatment adherence, and the improvement of positive mental health. The expectation is to generate patient empowerment and a reduction in the number of consultations and hospital admissions derived from some adverse effects of the pharmacological treatment.

### Potential Limitations and Implications

The main limitation of this study is that the sample is not randomized. Another limitation presented is the selection of patients included in the study according to the level of awareness and cognitive capacity as well as the ability to understand and use the MyPlan app.

The training nurse’s time availability to train and educate patients in their pharmacological treatment is another limitation to take into consideration.

### Conclusions

The results of this study will offer evidence of the effectiveness of a nursing pharmacological educational intervention that uses a free smartphone app. If its efficacy is demonstrated and the results are acceptable, it could mean an improvement in the care of patients operated for femur fractures and guide other training interventions in patients with other pathologies.

## Abbreviations

PI: principal investigator
mHealth: mobile health
WHO: World Health Organization

## Data Availability

The data confidentiality and availability disclaimer is included in the information sheet that is distributed to study participants and is explained to them prior to signing the informed consent form.
